# Continuous venous hemodialysis integrated to the ECMO circuit in critically ill patient with COVID-19, a case report in Morocco

**DOI:** 10.11604/pamj.supp.2020.35.141.25332

**Published:** 2020-08-10

**Authors:** Anass Mounir, Saara Lamghari, Amine Raja, Khalil Allali, Sara Chebbar, Basma Buri, Yasmine Mahdar, Chafik El Kettani, Benyounes Ramdani, Youssef Ettaoumi, Ghali Benouna, Lahoucine Barrou

**Affiliations:** 1 Department of Anesthesia and Critical Care, Ibn Rochd University Hospital Center, Casablanca, Morocco,; 2 Department of Nephrology And Hemodialysis, Ibn Rochd University Hospital Center, Casablanca, Morocco,; 3 Department Of Cardiovascular Surgery, Ibn Rochd University Hospital Center, Casablanca, Morocco,; 4 Department of Cardiology, Ibn Rochd University Hospital Center, Casablanca, Morocco

**Keywords:** COVID-19, ARDS, hemodialysis, ECMO

## Abstract

Novel coronavirus 2019 (COVID-19) is a severe respiratory infection leading to acute respiratory distress syndrome [ARDS] accounting for thousands of cases and deaths across the world. Several alternatives in treatment options have been assessed and used in this patient population. However, when mechanical ventilation and prone positioning are unsuccessful, venovenous extracorporeal membrane oxygenation [VV-ECMO] may be used. We present a case of a 62-year-old female, diabetic, admitted to the intensive care unit with fever, flu-like symptoms and a positive COVID-19 test. Ultimately, she worsened on mechanical ventilation and prone positioning and required VV-ECMO. The use of VV-ECMO in COVID-19 infected patients is still controversial. While some studies have shown a high mortality rate despite aggressive treatment, such as in our case, the lack of large sample size studies and treatment alternatives places healthcare providers against a wall without options in patients with severe refractory ARDS due to COVID-19.

## Introduction

The novel coronavirus 2019 (COVID-19) is a respiratory tract infection that has resulted in a pandemic, infecting more than 10,250,000 humans and claiming the lives of over 400,000 in less than six months [1]. The disease classically results in hypoxemic respiratory failure requiring oxygen supplementation using low and high delivery systems, as well as mechanical ventilation. However, when all these measures fail, options become very limited. One of these potential alternatives is the extracorporeal membrane oxygenation [ECMO]. Evidence on ECMO in COVID-19 patients remains controversial, as the immunological side effects of ECMO can further compromise the already debilitated immune system fighting COVID-19 [2]. We report a case of a COVID-19-positive patient who was managed with ECMO after no response to mechanical ventilation and prone positioning.

## Patients and Observation

**Figure 1:**
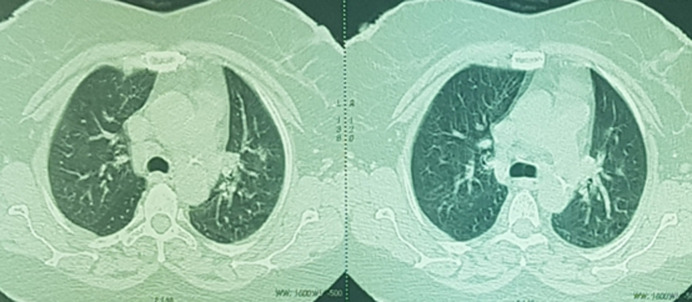
bilateral ground glass aspect on a chest scanner

**Figure 2:**
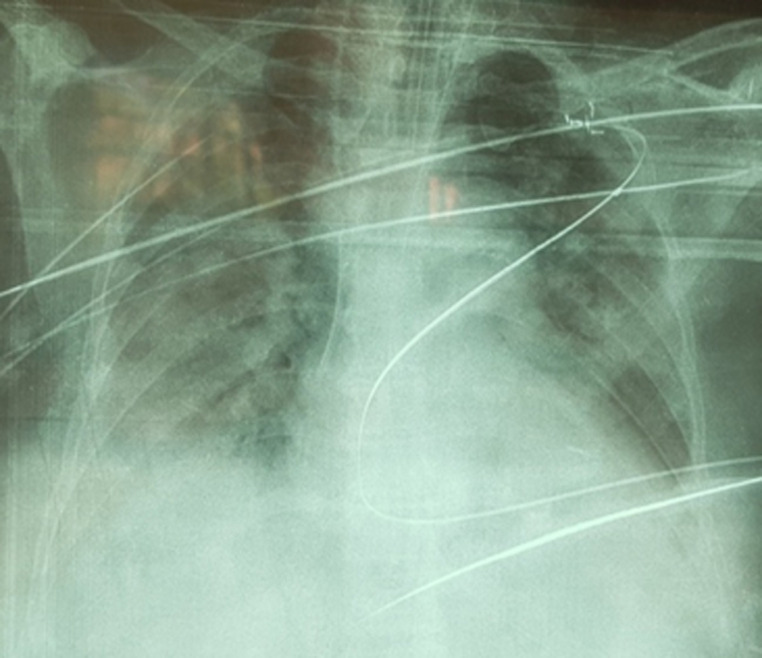
post-intubation chest X-ray

A 62-year-old diabetic woman on oral anti-diabetic drugs tested positive for severe acute respiratory syndrome coronavirus 2 [SARS-CoV-2]. The clinical examination on admission to our intensive care unit found a conscious patient [Glasgow Coma Scale at 15/15], feverish at 39°C, blood pressure at 110/70 mmHg, heart rate at 71 bpm, respiratory rate at 23 cpm, saturation at 86 on room air [96% under 10 L oxygen with non-rebreather] and a glycemia at 0.8 g/l; She was put on treatment: hydroxychloroquine, Corticotherapy, enoxaparin [0.4x2/day], 3^rd^ generation cephalosporin, paracetamol, Vitamine C and zinc sulfate. (Figure 1). The evolution was marked by the worsening of the respiratory state on Day 2 of hospitalization, the patient was intubated, ventilated, sedated and curarized under controlled assisted ventilation and protective mode [FiO_2_= 100%, RR = 22, PEEP = 10, Vt = 440ml], put under 1 mg/h of norepinephrine; post-intubation arterial blood gas showed respiratory acidosis and metabolic alkalosis with a ratio of partial pressure of arterial oxygen to the inspired oxygen fraction [P/F ratio] at 95 and a chest X-ray showed multiple alveolar opacities (Figure 2).

A Cocci Gram + was isolated in blood culture, hence the administration of antibiotics based on Vancomycin, Amikacin and imipenem. Despite careful management, the patient developed a severe acute respiratory distress syndrome [ARDS] with at arterial blood gas analysis pH = 7.43; PCO_2_= 57mmHg; PO_2_= 72mmHg; HCO_3_- = 36.9mEq/l and at P/F Ratio = 72. At D3 of hospitalization, in front of refractory hypoxemia and the failure of proneventilation, decision was to set up oxygenation by extracorporeal veinovenous membrane ECMO-VV [right femoro-jugular] under cover of heparin [10,000 IU/24h]; the internal jugular vein and the femoral vein were cannulated with heparin-coated cannulas. After one hour, an arterial blood gas reported mixed alkalosis with a P/F ratio of 128. She also received a dose [8 mg/kg] of Tocilizumab, an anti-interleukin-6 monoclonal antibody, to help control her cytokine storm on day 7, day 3 of ECMO. On Day 10, the patient became icteric with a distended abdomen and presented an oliguria, renal failure [urea = 1.56 g/l, creatinin = 24.2mg/l] and hyperkalemia at 5.4 mEq/l. Renal function continued to worsen, and patient was placed on continuous venous hemodialysis integrated to the ECMO circuit, with an improvement in the P/F ratio in ABG at 180 [vs 76]. On Day 24 of her hospitalization, Day 20 of ECMO, the hemodynamic status has become unstable, the patient was still anuric. She was stabilized with 5mg/h epinephrine. The ABG showed pH=6.86; PCO_2_=31mmHg; PO_2_=162mmHg; HCO_3_-=5.4mEq/l Subsequently, the patient was dialyzed, then she presented hypotension at 50/40 mm Hg and bradycardia at 45 bpm; then she was put on 12 mg/h of norepinephrine and 10 mg/h of epinephrine. A few hours later, the patient developed an asystole and then declared deceased.

## Discussion

ECMO refers to one of the extracorporeal life support technologies [ECLS], mainly adopted to partially or completely replace the cardiopulmonary function of patients in order to provide the oxygen needs of the organs and to try to take the time to treat primary diseases [3]. For the treatment of COVID-19, the health organization considers that oxygenation by extracorporeal membrane [ECMO] can act as a rescue treatment measure. Our patient is the first case of COVID-19 treated by ECMO in our department. After receiving active anti-infective treatment and invasive mechanical ventilation, oxygenation remained difficult to maintain. Following a discussion by multidisciplinary staff, the ECMO was successfully installed for almost 20 days before an organic dysfunction caused by severe ischemia and hypoxia. After ECMO treatment, the circulatory function tended to deteriorate with the onset of acute renal failure requiring continuous renal replacement therapy [4]. No definitive conclusion has yet been reached on the timing of the establishment of ECMO. The authors have consulted relevant research on ARDS caused by the influenza virus, among which Steimer *et al*. [5] consider that early treatment with ECMO can increase the survival rate of patients.

For our patient, the entire staff chose ECMO-VV because the patient only had pulmonary insufficiency without any underlying cardiac pathology, the value of the measured cardiac ejection fraction was around 67%. During ECMO management, close coagulation monitoring as well as blood gas analysis and chest x-ray are done regularly to prevent complications such as hemorrhage, thrombus and pneumothorax. Also, cardiac and pulmonary ultrasound can assess the daily lung condition and check the effect of treatment, in order to prepare for the early withdrawal of ECMO. Acute kidney injury [AKI] is a common complication in patients receiving ECMO due to multiple mechanisms, including underlying inflammation, hypoperfusion and exposure to nephrotoxins [6]. Like the majority of these patients who are hemodynamically unstable, the CRRT [continuous renal replacement therapy] method is the modality of choice. Although each type of extracorporeal assistance can be administered independently using separate venous access, many institutions integrate the CRRT device into the ECMO circuit [7]. The search for an efficient and safe method to connect an hemodialysis machine to the ECMO circuit is at the center of the current investigation. In addition, anecdotal evidence indicates that no COVID-19 patient with multiple organ failure survived ECMO [8].

## Conclusion

Extracorporeal membrane oxygenation remains an option for the treatment of patients with severe acute respiratory distress, in patients in certain age groups with minor comorbidities. It is a highly necessary procedure with high technicality and a trained team. ECMO will not replace mechanical ventilation. The combination of ECMO and CRRT seems to be a safe and effective technique that improves water balance and electrolyte disturbances. Prospective studies would be useful to determine the potential of this technique to improve outcomes in severe COVID-19 patients requiring hemodialysis.
